# Effect of Branched-Chain Amino Acid Supplementation on Recovery Following Acute Eccentric Exercise

**DOI:** 10.3390/nu10101389

**Published:** 2018-10-01

**Authors:** Trisha A. VanDusseldorp, Kurt A. Escobar, Kelly E. Johnson, Matthew T. Stratton, Terence Moriarty, Nathan Cole, James J. McCormick, Chad M. Kerksick, Roger A. Vaughan, Karol Dokladny, Len Kravitz, Christine M. Mermier

**Affiliations:** 1 Department of Exercise Science and Sport Management, Kennesaw State University, Kennesaw, GA 30144, USA; mstratt4@students.kennesaw.edu; 2 Department of Kinesiology, California State University Long Beach, Long Beach, CA 90840, USA; kurt.escobar@csulb.edu; 3 Department of Kinesiology, Coastal Carolina University, Conway, SC 29528, USA; kjohns10@coastal.edu; 4 Department of Health, Exercise and Sports Science, University of New Mexico, Albuquerque, NM 87131, USA; moria1ta@unm.edu (T.M.); ncole1@unm.edu (N.C.); aeneid@unm.edu (J.J.M.); lkravitz@unm.edu (L.K.); cmermier@unm.edu (C.M.M.); 5 School of Health Sciences, Lindenwood University, St. Charles, MO 63301, USA; ckerksick@lindenwood.edu; 6 Department of Exercise Science, Congdon School of Health Sciences, High Point University, High Point, NC 27268, USA; rvaughan@highpoint.edu; 7 Department of Internal Medicine, School of Medicine, University of New Mexico, Albuquerque, NM 87131, USA; kdokladny@salud.unm.edu

**Keywords:** BCAA, muscle damage, recovery, supplement, eccentric exercise, sports nutrition

## Abstract

This study investigated the effect of branched-chain amino acid (BCAA) supplementation on recovery from eccentric exercise. Twenty males ingested either a BCAA supplement or placebo (PLCB) prior to and following eccentric exercise. Creatine kinase (CK), vertical jump (VJ), maximal voluntary isometric contraction (MVIC), jump squat (JS) and perceived soreness were assessed. No significant (*p* > 0.05) group by time interaction effects were observed for CK, soreness, MVIC, VJ, or JS. CK concentrations were elevated above baseline (*p* < 0.001) in both groups at 4, 24, 48 and 72 hr, while CK was lower (*p* = 0.02) in the BCAA group at 48 hr compared to PLCB. Soreness increased significantly from baseline (*p* < 0.01) in both groups at all time-points; however, BCAA supplemented individuals reported less soreness (*p* < 0.01) at the 48 and 72 hr time-points. MVIC force output returned to baseline levels (*p* > 0.05) at 24, 48 and 72 hr for BCAA individuals. No significant difference between groups (*p* > 0.05) was detected for VJ or JS. BCAA supplementation may mitigate muscle soreness following muscle-damaging exercise. However, when consumed with a diet consisting of ~1.2 g/kg/day protein, the attenuation of muscular performance decrements or corresponding plasma CK levels are likely negligible.

## 1. Introduction

Skeletal muscle damage induced by resistance-based exercise is known to promote microdamage in muscle fibers, which may lead to temporary increased passive tension, delayed onset muscle soreness (DOMS), decrements in strength and force production, and increased efflux of intramuscular proteins into the blood [1]. The degree of damage and discomfort may be compounded over time and persist chronically, especially in individuals frequently engaging in vigorous exercise or those completing an overreaching phase [1,2]. As such, nutritional strategies have been proposed to mitigate the negative effects that may be experienced following strenuous resistance exercise. Protein and amino acid supplements, including branched-chain amino acids (BCAA), have been considered a potentially efficacious dietary intervention [3,4,5]. BCAAs (i.e., leucine, isoleucine and valine) are distinct among essential amino acids in that they are extrahepatically metabolized in skeletal muscle [6,7]. It has been suggested that BCAA supplementation may reduce protein degradation and/or muscle enzyme release [2,3,8], decrease skeletal muscle damage in response to intense resistance exercise [9,10,11], reduce feelings of soreness [12], mitigate central fatigue [13,14] and promote subsequent recovery of muscle function [10,15]; however, these findings remain inconclusive at present [15,16,17]. While underlying mechanisms remain unclear [2,18], BCAA supplementation is a popular practice among recreational exercisers and athletes [8,19,20] and continues to garner significant research interest. For example, Howatson et al. [10] examined the impact of 12 days of two daily doses of 10 grams (g) of BCAA or placebo in trained males who completed a workout consisting of 100 drop-jumps. In comparison to placebo, plasma creatine kinase (CK), perceived soreness and force production were all improved for the first 24 hr while soreness remained significantly lower up to 48 hr after damaging exercise in the BCAA supplemented group. No differences were noted for vertical jump. In addition, Sharp and colleagues [11] supplemented eight recreationally active men with either a placebo or 6 g of BCAA for three weeks and reported a reduction in CK levels 12 and 36 hr after completing two days of intense resistance exercise. Jackman and investigators [15] reported that compared to placebo treatment, 29.2 g of BCAA per day resulted in decreased DOMS at 48 and 72 hr in 24 non-resistance trained males after unilateral eccentric exercise. However, no differences in percent change for electrically stimulated maximal isometric force of the quadriceps, plasma CK, myoglobin and interleukin-6 response between groups post-exercise were observed. Further, Foure et al. [16] found that muscle soreness and MVIC in 26 recreationally active men were not affected by 0.1 g/kg of BCAA ingested pre- and post-damaging neuromuscular electrostimulation exercise.

Results surrounding the ability of BCAAs to favorably impact recovery from damaging exercise are mixed. While a multitude of reasons for these inconsistencies exist, controlling for dietary protein intake seems to be an area that previous research has not adequately considered. In this respect, Howatson et al. [10] reported significant improvements in force production, circulating CK levels and perceived soreness, but daily protein intake was not controlled which could have resulted in discrepancies in overall amino acid intake. Moreover, Sharp and colleagues [11] also reported positive outcomes for BCAA supplementation, but individuals who were consuming a daily protein intake above the recommended daily allowance (0.8 g/kg/day) were excluded from the study. Foure et al. [16] reported no difference between BCAA and placebo in soreness and force production. Of note, participants in the Foure et al. investigation who were supplemented with BCAAs consumed significantly greater quantities of daily protein on supplementation days, 1.5 g/kg/day, while the protein intake for the placebo group averaged 1.07 g/kg/day. Finally, Jackman et al. [15] reported only reductions in soreness when individuals were supplemented with 29.2 g of BCAAs per day or a placebo after a damaging bout of exercise. Notably, daily protein intake in the Jackman study was controlled at 1.5 g/kg/day for subjects in both BCAA and placebo groups which may have influenced their final outcomes. Therefore, it seems possible that when BCAA supplementation is provided while daily protein intake is not already at recommended levels [21,22], the potential for BCAA administration to afford any additional impact may be improved.

As it stands, the inconsistencies documented in numerous investigations following damaging resistance exercise and subsequent recovery prevents any conclusive inferences regarding the efficacy of BCAA supplementation. Curiously, BCAA supplementation has still been associated with reduced perceived soreness following intense resistance exercise [5,15,23], though mechanisms explaining the relationship between BCAA ingestion and perception of muscle soreness are not well established. Moreover, current evidence suggests the attenuation of DOMS [5,15,24], as well as efflux of biochemical markers of muscle damage in individuals supplementing with BCAA [5,10,11], do not necessarily occur with a concomitant enhancement of muscle function recovery [4,5,15]. Adding to this complexity are the discrepancies in training state of study participants, damaging exercise protocols and overall protein intake employed within the limited number of studies completed. Finally, the population most apt to supplement with BCAA to attenuate the negative effects of intense resistance exercise are resistance training individuals who are likely already ingesting a moderate protein intake (1.4–2.0 g/kg/day) [21], thus potentially making exogenous BCAA consumption superfluous. For these reasons, the present study aimed to investigate the effects of BCAA supplementation on markers of muscle damage and recovery of muscle function in resistance trained males while adhering to a protein intake slightly lower than the recommended range for resistance training individuals.

## 2. Materials and Methods

### 2.1. Participants

Twenty young, resistance-trained (RT) males (age 22.3 ± 1.5 year, height 175.4 ± 6.7 cm and body mass 86.4 ± 15.6 kg) were recruited for the study. The present study was approved by the institution’s Human Research Review Committee. Participants were made aware of all procedures’ risks and benefits, gave written consent and completed health history, diet history and physical activity questionnaires. All participants had several years (5.3 ± 2.5 year) of resistance exercise training experience, with an average self-reported training time of 7.3 ± 2.1 hr per week. Participants were excluded if they were consuming creatine (within the past six months) and certain medications (non-steroidal anti-inflammatory or steroidal drugs). Individuals consuming protein supplements (e.g., whey, casein) were asked to refrain from taking these supplements following enrollment into the study. Individuals utilizing treatments such as cryotherapy or massage, past or current smokers, those without at least one year of current resistance training experience and participants who had completed high volumes of unaccustomed lower-body resistance-based exercise in the last six months (to control for the repeated bout effect) [9] were also excluded from the study. Further, all included participants refrained from exercise and alcohol consumption 48 hr prior to testing and throughout the entire testing period and caffeine 12 hr prior to each visit. Participant characteristics are shown in Table 1.

### 2.2. Experimental Design

**Figure 1:**
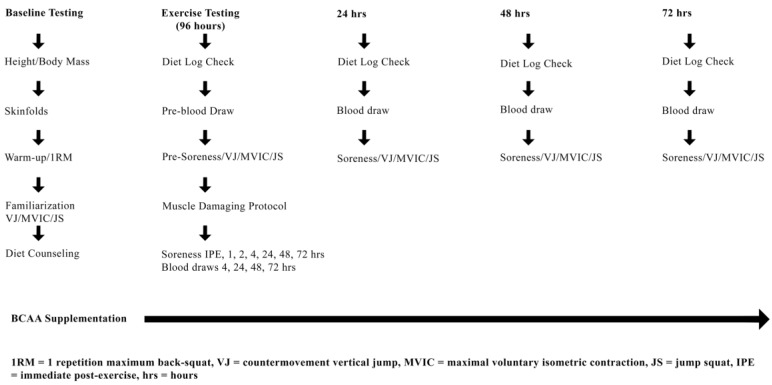
Experimental design overview. Participants were supplemented for 8 days with either BCAA or placebo following baseline assessment. Participants returned to the lab 96 hr following baseline assessment.

Using a randomized, double-blind, placebo-controlled research design, participants were enrolled into either a BCAA (MusclePharm BCAA 3:1:2 watermelon powder) or PLCB group (color and flavor matched maltodextrin) and performed one experimental muscle-damaging exercise trial. All participants were thoroughly familiarized with the study design; specifically the diet and physical activity log requirements, timing and procedures of blood collection, 1RM protocol, performance measures (i.e., vertical jump (VJ), maximal voluntary isometric contraction (MVIC), jump squat (JS)) and supplementation regimen. Figure 1 depicts the overall study design. 

### 2.3. Baseline Testing: Enrollment, 1RM, Familiarization and Dietary Counseling

On the day of baseline testing, following consent and determination of involvement, participants first had their height and body mass measured and their body composition assessed using the skinfold technique. Participants were then asked to complete a self-selected ten-minute warm-up followed by Smith machine 1RM assessment. Following 1RM assessment, participants were asked to rest for ten-minutes and then complete a thorough familiarization of the countermovement VJ, MVIC of the quadriceps at 120 degrees of knee flexion (leg flexion angle of 60° below the horizontal plane), and 40% 1RM JS performance in order to eliminate any learning effects on test performance during data collection. Test-retest reliability was determined for all performance variables during the familiarization sessions and was deemed acceptable if they were greater or equal to *r* = 0.90. Such reliability has been shown to be essential for experimental sensitivity to nutritional interventions [25]. Following the completion of 1RM assessment and familiarization of performance assessments, participants were counseled on supplementation, dietary requirements, and dietary tracking. Participants were asked to return 96 hr later to complete the muscle damaging protocol. 

### 2.4. Anthropometric Measurements

Measurements of height, body mass and body fat percentage were obtained to characterize the participants. Body mass was measured via a Tanita electronic scale (Model #3101, Arlington Heights, IL, USA) to the nearest 0.1 kg. Two skinfold measurements on the right side of the body were obtained from three sites (chest, abdomen and thigh) in serial fashion by the same investigator utilizing Lange Calipers (Cambridge Scientific Industries, Cambridge, MD, USA). Skinfold thickness was based on the average of the two trials. If the two skinfold measurements for a particular site varied by more than 0.5 millimeters (mm), the technician obtained a third measurement and the mean value of the two closest measurements was used. Body density was then calculated according to the Jackson and Pollock 3-site prediction equation [26] and percent body fat was estimated according to Siri [27]. The ethnic make-up of our participants consisted of sixteen Caucasian and four Hispanic males. Currently, no population specific-equation to estimate percent body fat for Hispanics exists. Therefore, the Siri equation was used for all participants.

### 2.5. One-Repetition Maximum (1RM)

Smith machine (Pro-Elite Strength Systems, Salt Lake City, UT, USA) back squat 1RM was determined according to methods previously described [28]. Following a ten-minute standardized, dynamic warm-up, each participant first performed a warm-up set of 8–10 repetitions at a light-weight (∼50% of their estimated (est) 1RM). A second warm-up consisted of a set of 3–5 repetitions with a moderate weight (∼75% of 1RM_est_) and a third warm-up included 1–3 repetitions with a heavy weight (∼90% of 1RM_est_). After the warm-up, each participant’s 1RM was tested by increasing the load during consecutive trials until the participants were unable to perform a proper lift using correct technique (90 degrees of knee flexion). The 1RM test was determined by 4–6 sets of one repetition, with 3–5 min of rest between attempts. Spotters were present to provide verbal encouragement and spotting to ensure safety of the participants.

### 2.6. Supplementation, Diet and Diet Tracking

Participants ingested 0.22 g/kg/day of BCAA (MusclePharm, Denver, CO; 7.16 g = 3 g leucine, 1 g isoleucine, 2 g valine) or maltodextrin (PLCB) in dry-powder form mixed with water (~175–350 mL) for a total of 8 days following baseline assessment. The supplements were separated into one morning and one evening dose per day. On the fifth day of supplementation, participants returned to the laboratory and completed the muscle-damaging squat-exercise protocol. Participants were instructed to consume their supplement prior to completing the exercise visit as well as follow-up visits. A research team member provided each participant with specific verbal and written directions and procedures for reporting a detailed dietary intake, including information on how to record portions using household measures, preparation technique and nutrient content descriptors (e.g., reduced-fat, light). During the entire experimental period (8 days), participants recorded their food intake using a paper food log and were instructed, if needed, by a registered dietician on how to achieve a protein intake of 1.2 g/kg/day of body mass. Further, participants were instructed to follow their normal total calorie intake throughout the duration of the study. Participant’s dietary logs were then entered into MyFitnessPal (Under Armour, Baltimore, MD, USA) in order to calculate macronutrient and calorie intake.

### 2.7. Experimental Protocol (Muscle Damaging Exercise Visit)

Participants were asked to return to the laboratory 96 hr following baseline testing to undergo a muscle damaging exercise protocol. Participant’s dietary logs were checked for protein compliance upon arrival, followed by a pre-exercise blood collection, rating of perceived soreness, and assessments of VJ, MVIC, and 40% 1RM jump squat. Participants then completed the muscle-damaging squat exercise protocol. Following completion of the exercise protocol, participants rated their current state of soreness and completed all measures of exercise-performance immediately post-exercise (IPE), 1, 2, 4, 24, 48 and 72 hr post-squat exercise. Participant’s blood was collected 4, 24, 48 and 72 hr post-exercise. The same trained researcher was used for the collection of each participant’s performance measures.

### 2.8. Muscle Damaging Protocol

A standardized bout of resistance exercise involving ten sets of eight repetitions at 70% 1RM squats using a Smith machine (Pro-Elite Strength Systems, Salt Lake City, UT) was completed by all participants. Each repetition throughout the squat protocol consisted of a four-second lowering and a one-second raising of the load monitored by a metronome and spotter. A stopwatch was started following the last repetition of each set and participants were given three minutes of rest between all squat sets. Following completion of the squat protocol, participants then completed five sets of 20 consecutive (10 each leg) body-weight split jump repetitions with two minutes of rest between each set.

### 2.9. Markers of Muscle Performance and Soreness

#### 2.9.1. Vertical Jump

Maximum countermovement VJ was assessed using a Vertec device (Perform Better, West Warwick, RI, USA). Participants were instructed to stand with their feet flat and shoulder-width apart on the ground directly beneath the Vertec. Participants were then instructed to reach up as high as possible with a single arm to measure standing reach height, defined as the height of the highest Vertec vane a participant could reach. Participants were then asked to complete a countermovement jump using both feet, while reaching up to touch the highest reachable vane. Each participant’s standing reach height was then subtracted from the highest vane touched after the countermovement jump. Participants were allowed three jumps, with the highest jump recorded and used for statistical analysis.

#### 2.9.2. Maximal Voluntary Isometric Contraction

Maximum voluntary isometric (MVIC) strength of quadriceps of the dominant limb was measured by a dynamometer (Biodex Medical Systems, System 4, Shirley, NY, USA). Participants sat upright with the chair’s backrest inclined to 85°, with their knee placed in 120° of flexion (leg flexion angle of 60° below the horizontal plane) [29]. The axis (i.e., lateral epicondyle of the femur) of the knee was aligned with the rotational axis of the dynamometer. For the test, participants were asked to perform a MVIC of the quadriceps muscles for five seconds and then rest for one minute. Participants completed this cycle three times and the peak torque value (newton-meters) was recorded.

#### 2.9.3. Jump Squat

Jump squat peak power (PP) was determined by taking the better of two maximal effort JS at 40% of each participant’s 1RM with one minute of rest. All JS were performed using a Smith squat rack (Pro-Elite Strength Systems, Salt Lake City, UT, USA). The countermovement depth during the JS was self-selected as previously described [30,31]. During the JS, subjects were asked to hold a bar across their shoulders and keep constant downward pressure on the bar so that it would not move independently of the body. Power production was determined via a Tendo Power Analyzer (TENDO PSA 310; Irmo, SC, USA).

#### 2.9.4. Perceived Soreness

Participants were asked to evaluate their perceived level of muscle soreness using a visual analog scale. Soreness was assessed along a 10 cm scale (0 cm = no soreness, 10 cm = extreme soreness) for each time point (pre-exercise, IPE, 1, 2, 4, 24, 48 and 72 hr post-squat exercise) by drawing a line perpendicular to the continuum line extending from 0 to 10 cm. Soreness was evaluated by measuring the distance of each mark from 0 and rounded up to the nearest one-tenth of a centimeter [11].

### 2.10. Blood Sampling and Analysis

Venous blood was collected pre-squat exercise, 4, 24, 48 and 72 hr post-exercise for the collection of plasma. As an indirect marker of muscle damage, plasma concentrations of creatine kinase (CK) were determined in duplicate using an enzymatic assay (Pointe Scientific, Canton, MI, USA) and a spectrophotometer (Beckman Coulter, DU-520, Fullerton, CA, USA) at a wavelength of 340 nanometers (nm).

### 2.11. Statistical Analysis

Statistical tests were conducted in R (version: 3.2.2; R Foundation for Statistical Computing; Vienna, Austria) using the ‘afex’ package (version 0.16-1). Using MVIC data generated, a post-hoc power analysis was completed. An estimated effect (d) of the interaction effect revealed a moderate effect of 0.61. At a sample size of 10 participants per group and delta responses for both the PLA and BCAA group at the 24 hr post-exercise time-point resulted in a statistical power of 0.523. Separate mixed-effects (within-between) factorial ANOVAs (group × time) were used to assess the main and interaction effects for each reported dependent variable. Post-hoc pairwise comparisons were then used to investigate group differences across individual time-points with the Bonferroni adjustment applied to correct for multiple comparisons. ANOVA models were evaluated for compliance with underlying model assumptions. Assumptions of sphericity were tested using Mauchly’s test of sphericity and violations were corrected using the Greenhouse-Geisser correction factor. Unpaired t-tests were used to determine differences in years of previous resistance training experience, body mass, average study protein and calorie intake, and 1RM back squat for BCAA and PLCB groups. The threshold for statistical significance was set *a priori* at *p* ≤ 0.05 for all analyses.

## 3. Results

No significant differences in previous resistance training experience (*p* = 0.55), 1RM back squat (*p* = 0.80) and body mass (*p* = 0.95) were found at baseline between groups. Further, there were no significant differences in protein intake (g/kg/day; *p* = 0.42) and total calories consumed per day (*p* = 0.57). All subjects successfully completed the 80 eccentric squats and 100 body-weight split jumps (50 each leg). Four individuals (two from each group) decreased the weight by 10 lbs. (~4.55 kg) in order to complete the eccentric squat exercise protocol (*n* = 2: set 6; *n* = 1: set 8; *n* = 1: set 9). All performance measures (VJ, JS, MVIC), soreness ratings, and creatine kinase showed time effects (*p* < 0.05) for both PLCB and BCAA groups indicating that the eccentric exercise protocol effectively induced muscle damage.

### 3.1. Muscular Performance: Vertical Jump

**Figure 2:**
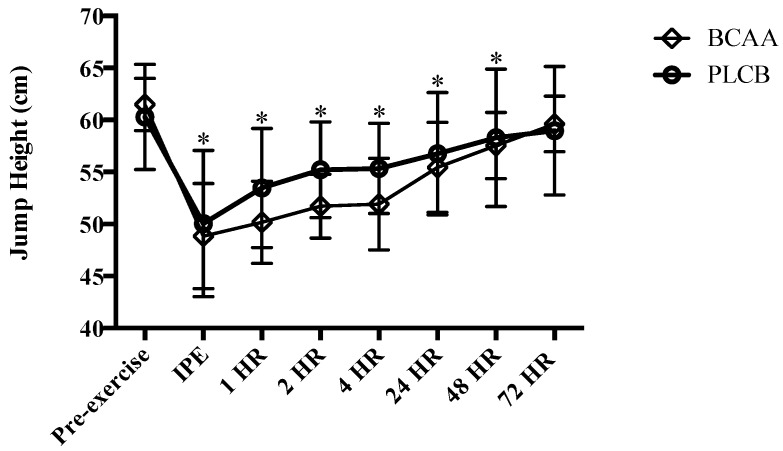
Mean (± standard deviation) jump height (cm; centimeters) pre-eccentric exercise, immediate post (IPE), 1, 2, 4, 24, 48 and 72 hours (HR) for resistance trained men supplementing with branched-chain amino acids (BCAA) or placebo (PLCB) (*n* = 20). * = significantly different from pre-exercise (*p* < 0.05) for both BCAA and PLCB.

Both groups demonstrated similar (*p* > 0.05) VJ height prior to the eccentric exercise protocol (pre-exercise) (BCAA = 68.71 ± 2.90 cm; PLCB = 69.60 ± 5.87 cm). Vertical jump height was significantly lower for both BCAA and PLCB groups IPE, 1, 2, 4, 24 and 48 hr post-eccentric exercise (*p* < 0.05); however, there were no group or interaction effects for vertical jump performance (Figure 2).

### 3.2. Muscular Performance: Maximal Voluntary Isometric Contraction

**Figure 3:**
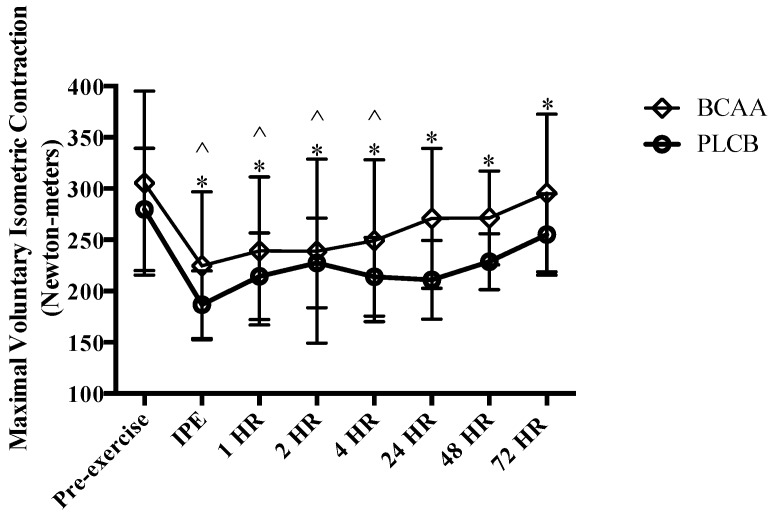
Mean (± standard deviation) force production (Newton-meters) pre-eccentric exercise, immediate post (IPE), 1, 2, 4, 24, 48 and 72 hours (HR) for resistance trained men supplementing with branched-chain amino acids (BCAA) or placebo (PLCB) (*n* = 20). * = significantly different from pre-exercise (*p* < 0.05) for PLCB. ^ = significantly different from pre-exercise (*p* < 0.05) for BCAA.

Both groups demonstrated similar (*p* > 0.05) force output prior to the eccentric exercise protocol (pre-exercise) (BCAA = 305.3 ± 89.7; PLCB = 279.7 ± 59.9 Nm (Newton-meters)). Maximal voluntary isometric force output was significantly lower at all post-exercise time points for the PLCB group, while the BCAA group only displayed significantly lower values IPE, 1, 2 and 4 hr post-eccentric exercise (*p* < 0.05). Force output was not significantly different from baseline measures at 24 hr (*p* = 0.18; BCAA: 270.8 ± 68.8 Nm), 48 hr (*p* = 0.11; BCAA: 271.4 ± 45.8 Nm), or 72 hr (*p* = 0.21; BCAA: 295.5 ± 77.1 Nm) and no significant group-by-time effect was observed at any of the time-points (*p* > 0.05) (Figure 3). 

### 3.3. Muscular Performance: Jump Squat

**Figure 4:**
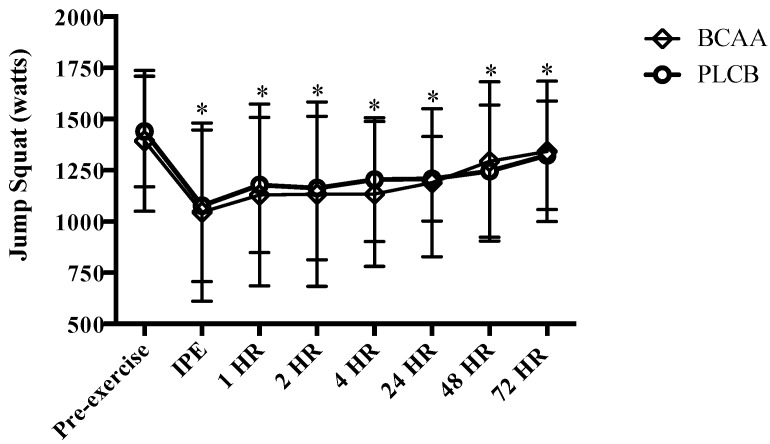
Mean (± standard deviation) peak power output (watts) pre-eccentric exercise, immediate post (IPE), 1, 2, 4, 24, 48 and 72 hours (HR) for resistance trained men supplementing with branched-chain amino acids (BCAA) or placebo (PLCB) (*n* = 20). * = significantly different from pre-exercise (*p* < 0.05) for both BCAA and PLCB.

Both groups demonstrated similar (*p* > 0.05) peak power output as measured by the 40% 1RM JS prior to the eccentric exercise protocol (pre-exercise) (BCAA = 1392.9 ± 344.1; PLCB = 1439.1 ± 270.5 watts). Peak power output was significantly lower for both BCAA and PLCB groups IPE, 1, 2, 4, 24, 48 and 72 hr following eccentric exercise (*p* < 0.05); however, there were no group or interaction effects for JS performance (Figure 4). 

### 3.4. Muscle Soreness

**Figure 5:**
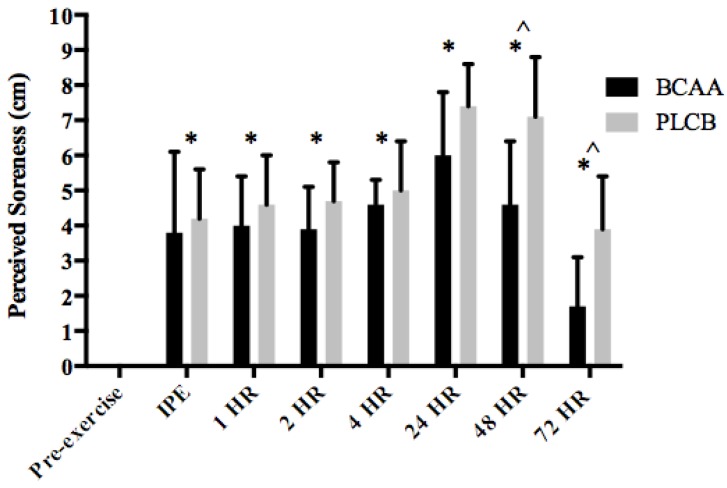
Mean (± standard deviation) perceived soreness rating (cm; centimeters) pre-eccentric exercise, immediate post (IPE), 1, 2, 4, 24, 48 and 72 hours (HR) for resistance trained men supplementing with branched-chain amino acids (BCAA) or placebo (PLCB) (*n* = 20). * = significantly different from pre-exercise for both BCAA and PLCB (*p* < 0.05); ^ = significantly different from PLCB group (*p* < 0.05).

Both groups demonstrated similar pre-exercise perceived soreness ratings (BCAA = 0 ± 0; PLCB= 0 ± 0 cm). Perceived soreness was significantly elevated for both BCAA and PLCB groups IPE, 1, 2, 4, 24, 48 and 72 hr post-eccentric exercise (*p* < 0.05), however the BCAA group reported significantly less soreness (*p* < 0.01) at 48 hr (BCAA: 4.59 ± 1.42; PLCB: 7.14 ± 1.65 cm) and 72 hr post-exercise (BCAA: 1.38 ± 1.83 cm; PLCB: 3.90 ± 1.52 cm) (Figure 5).

### 3.5. Blood Parameter: Creatine Kinase

**Figure 6:**
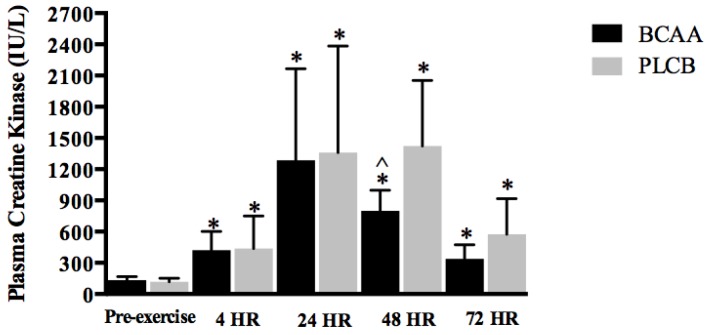
Mean (± standard deviation) plasma creatine kinase (IU/L; international units per liter) pre-eccentric exercise, 4, 24, 48 and 72 hours (HR) for resistance trained men supplementing with branched-chain amino acids (BCAA) or placebo (PLCB) (*n* = 20). * = significantly different from pre-exercise (*p* < 0.001); ^ = significantly different from PLCB group (*p* = 0.02).

Figure 6 displays the changes in plasma CK activity over the course of the experimental period. There were no significant differences between groups at pre-exercise (*p* > 0.05; BCAA = 134.5 ± 34.0; PLCB = 117.3 ± 34.8 IU/L). Plasma CK concentrations were significantly elevated above baseline (*p* < 0.001) in both BCAA and PLA groups at 4, 24, 48 and 72 hr post-exercise. While no significant group-by-time effect was detected for plasma CK (*p* = 0.10), plasma CK levels were significantly lower for the BCAA group at 48 hr post-exercise (*p* = 0.02; BCAA: 799.2 ± 197.6; PLCB: 1422.9 ± 630.8 IU/L).

## 4. Discussion

The aim of the present study was to examine the effect of BCAA supplementation on indices of muscle damage in resistance-trained men consuming a strict protein diet intended to provide a protein intake of 1.2 g/kg/day. To date, this is the first study examining the potential of a BCAA dose, normalized to body mass, to mitigate damage and enhance recovery following acute squat eccentric exercise in resistance-trained males undergoing strict dietary control. The ability of our eccentric squat exercise protocol to evoke skeletal muscle damage was indirectly evaluated by post-exercise changes in power production, isometric force loss, plasma CK concentrations and participants’ soreness ratings. It is evident from the significant time effects [10,32,33] and magnitude of response for each of these indices that muscle damage was inflicted [34], thus allowing us to sufficiently study recovery up to 72 hr post-exercise. To our knowledge, this eccentric exercise protocol has never been used in previous research; however, our data support the efficacy of this protocol to induce muscle damage using a manner of training that may be implemented into a resistance trainee’s program.

Proficient recovery procedures following strenuous, muscle-damaging exercise sessions are important for supporting training-induced adaptation and promoting quality subsequent exercise sessions. Amino acids have been shown to increase protein synthesis in the post-exercise period [35,36] and their consumption by athletes and recreationally trained individuals is a common practice to promote recovery. Research suggests greater protein synthetic rates and amino acid availability reduces damage to myofibrillar and cytoskeletal proteins, thereby helping to preserve force production abilities [36,37].

The muscle force generating capabilities during the recovery period following eccentric exercise have been suggested to be one of the most reliable indices of muscle damage due to the relationship between muscle force and muscle function [34]. Therefore, we chose to examine the effect of BCAA supplementation on recovery of muscle force production during an MVIC of the dominant leg quadriceps muscles as well as to evaluate recovery of more ballistic movements. While we found no differences in vertical jump height or loaded jump squat peak power between groups, MVIC force recovery was significantly recovered at 24, 48 and 72 hr post-exercise time-points for the BCAA group. These findings are similar to that of Howatson and colleagues [10] who reported a significantly lower decrement in MVIC force production and increased force production recovery in trained males supplementing with 20 g per day (10 g twice per day of BCAA, an additional 20 g bolus one hour pre-exercise, and another 20 gram bolus immediately post-exercise). However, the participants’ vertical jump height was unaffected. On the other hand, Foure et al. [16] reported 7 g of BCAA per day had no effect on recovery of quadriceps MVIC in recreationally trained males following muscle damage. Likewise, in a study by Jackman et al. [15], no differences were detected in force producing capabilities in untrained males supplementing with 29.3 g of BCAA per day as measured by MVIC. Kirby and coworkers [38] examined the effect of 250 mg/kg of the BCAA leucine on recovery of force production and vertical jump height. While no differences in jump height were detected, leucine attenuated mean peak force decrements across all post-exercise time points (up to 96 hr) in untrained males. Similar results from two cross-over investigations examining BCAA supplementation on recovery of muscle function in untrained individuals reported favorable outcomes of muscle function assessments [39,40]. However, these findings may be influenced by the repeated bout phenomena and should be considered a limitation [41,42]. While MVIC testing is a popular, valid and reliable measure of muscle function and recovery [43], it is important to note that the isometric movement associated with MVIC testing is distinctly different from several types of athletic performance movements. Though our data and others’ work [10,12] suggest enhanced recovery of force production in individuals consuming BCAAs, no studies to date provide evidence that BCAA supplementation supports recovery of more ballistic and functional movements [10,17,38].

While our data suggest a minor impact of BCAA supplementation on muscle function during recovery from muscle damaging exercise, the BCAA group also reported significantly less soreness 48 and 72 hr post-exercise. This is in agreement with previous work [15,44] and suggests the relationship between muscular function and soreness is not necessarily inversely related. Even though no improvement in muscle function was detected, untrained men consuming a diet consisting of 1.5 g/kg/day of protein and four doses of 7.3 g BCAA per day reported significantly less soreness 72 hr post eccentric exercise [15]. Similar findings by Howatson et al. [10] and Shimomura et al. [39] were reported 24 and 48 hr post resistance-based muscle damaging exercise in individuals consuming 20 g per day BCAA and 100 mg/kg body mass, respectively. Further, there is evidence that mixed amino acid supplementation decreases perception of muscle soreness by 30% when ingested during recovery from muscle-damaging exercise [5]. The mechanism(s) by which BCAA supplementation decreases muscle soreness cannot be deduced by our experimental study design; however, it has been suggested that enhanced glutamine production from BCAA degradation may be partly responsible for these observations [45]. Intense eccentric exercise results in significant increases in markers of inflammation. Previous research suggests these increases in inflammation heighten the sensitivity of muscle nociceptors [46] and correlates with increased feelings of soreness [46,47]. Upon consumption, transamination of some BCAAs to glutamate in order to synthesize glutamine may occur. In turn, glutamine may be consumed by inflammatory cells under inflammatory conditions [48]. Nicastro and colleagues [48] suggest that BCAAs decrease the inflammatory status of damaged muscle through increased availability of amino acids as substrates for immune cells, glutamine in particular; however, further research is needed to confirm this hypothesis.

We examined the effect of BCAA supplementation on one surrogate marker of muscle damage, CK, during the recovery period. Efflux of CK into the blood is indicative of sarcolemma disruption [34]. Several studies suggest an effect of amino acid supplementation on CK efflux following muscle damaging exercise [8,10,12,39]. Plasma CK following our eccentric squat protocol was significantly elevated from pre-exercise levels in both groups; however, resistance trained men supplementing with BCAA demonstrated significantly lower values 48 hr post-exercise when compared to the placebo group. Though non-significant, our data show the BCAA group’s plasma CK levels were lower at all time-points when compared to those of the placebo group and significantly lower at 48 hr. While we do not feel the 48 hr reduction in CK is physiologically relevant to the BCAA supplementation, numerous studies suggest amino acid supplements are effective at reducing CK efflux caused by damaging endurance or resistance exercise [8,10,39,49]. Following an acute bout of muscle damaging exercise, Howatson et al. [50] described significant reductions in plasma CK concentrations in well-trained, competitive rugby and national football players supplementing with 10 g of BCAA twice daily, with an additional 20 g prior to and immediately post-exercise. Similarly, 0.4 g/kg/day of a supplement (Big One, Professional Dietetics, Milan, Italy; 3 divided doses daily) containing 13 amino acids, including the BCAAs, attenuated plasma CK increases after one week of an overreaching program [51]. A report from Shimomura et al. [39] investigating untrained women completing body weight squats and consuming 100 mg/kg BCAA prior to exercise demonstrated lower CK values compared to that of a placebo group. Coombes and McNaughton [8] showed that increases in serum CK concentrations following 120 min cycling at 70% of each participant’s maximal oxygen uptake were significantly lower for the males consuming 12 g per day of BCAA with an additional 20 g consumed immediately pre- and post-cycling exercise. Data from Ohtani and colleagues [52] demonstrated that 6.6 g/day of a mixed amino acid supplement (BCAA, arginine, glutamine) attenuated increases in serum CK activity during recovery from strenuous long-distance running. However, following marathon performance, 5 g/day of BCAA did not attenuate changes in myoglobin, another indirect biochemical marker of muscle injury (e.g., sarcolemma disruption) [17]. In the only study to date examining leucine supplementation alone on indirect measures of muscle damage, 250 mg/kg (30 min before, during and immediately post-exercise and the morning of each recovery day following exercise) was ineffective at attenuating increases in CK and myoglobin in untrained males who completed an acute bout of muscle damaging resistive exercise [38]. While data exists to suggest BCAA supplements reduce the efflux of CK following a damaging or strenuous bout of exercise, the mechanism by which BCAAs assist in repairing/preserving the muscle sarcolemma membrane has yet to be elucidated. Results from several studies demonstrate that muscle cells have an efficient sarcolemma repair system to mediate response to local damage [34]. Small tears within the sarcolemma are typically sealed within seconds in healthy muscle. The protein dysferlin is a chief mediator of membrane resealing in muscle [53]. Future research studies should be developed to examine the relationship between amino acid availability and sarcolemma remodeling.

Although we feel this study has substantial external validity, results should be interpreted with caution as we were unable to use a cross-over study design due to the repeated bout effect associated with eccentric exercise [54]. Also, while we included strict dietary instructions, we were unable to prepare and administer meals for participants or control the amino acid content of each participant’s diet. Though the recommended protein intake for this study was lower than the recommended intake for resistance training males, we acknowledge the protein intake may have been sufficient to promote recovery as well as understand the error associated with self-reporting dietary intake. Future research studies should examine the effect of BCAA supplementation on recovery of relevant ballistic and athletic movements during the post-exercise period, as well as maximal strength assessments. 

## 5. Conclusions

The results of this investigation demonstrate that supplementing a controlled diet of 1.2 g/kg/day of protein with 0.22 g/kg body mass/day of BCAA for eight days results in decreased perceptions of soreness in resistance-trained individuals with several years of experience. However, BCAA supplementation in this fashion provides a minimal protective effect on attenuating other indirect makers of muscle damage following eccentric-based resistance exercise. While our results suggest BCAA may aid in the maintenance of isometric muscle function following muscle damage, this ergogenic effect may be trivial as there was no effect on dynamic measures of muscle function. Since the majority of recreational individuals and athletes will most likely engage in subsequent exercise sessions that consist of dynamic movements rather than isometric contractions, the ability of BCAA supplementation to maintain force output during isometric contractions lacks applicability. Therefore, when consumed with a diet consisting of 1.2 g/kg/day protein and presumably higher daily protein intakes, it appears BCAA effects on muscle recovery are negligible.

## Figures and Tables

**Table 1:** Participant characteristics.

Characteristic	BCAA	PLCB
Participant #	10	10
Age (yr)	23.0 ± 1.2	21.5 ± 1.5
Height (cm)	177.6 ± 7.1	173.2 ± 6.2
Body Mass (kg)	86.6 ± 15.2	86.2 ± 16.8
Body Fat%	12.3 ± 3.8	11.7 ± 4.3
1RM Squat (kg)	154.8 ± 31.7	155.0 ± 32.0
RT Experience (yr)	5.6 ± 2.3	5.0 ± 1.9
RT Experience (hrs/week)	7.00 ± 2.30	7.65 ± 2.05
Protein Intake (g/kg/d)	1.29 ± 0.12	1.25 ± 0.09
Average Calorie Intake (kcal)	2555 ± 324	2638 ± 309

All values are mean ± SD. # = number of participants, yr = years, cm = centimeters, kg = kilograms, 1RM = one repetition maximum, RT = resistance training, BCAA= branched-chain amino acid, PLCB= placebo, g/kg/d = grams/kilogram/day during study enrollment, kcal= calorie intake per day during study enrollment, hrs/week = hours per week.

## References

[1] Howatson G., van Someren K.A. (2008). The prevention and treatment of exercise-induced muscle damage. Sports Med..

[2] Da Luz C.R., Nicastro H., Zanchi N.E., Chaves D.F., Lancha A.H. (2011). Potential therapeutic effects of branched-chain amino acids supplementation on resistance exercise-based muscle damage in humans. J. Int. Soc. Sports Nutr..

[3] Kreider R.B., Wilborn C.D., Taylor L., Campbell B., Almada A.L., Collins R., Cooke M., Earnest C.P., Greenwood M., Kalman D.S. (2010). ISSN exercise & sport nutrition review: Research & recommendations. J. Int. Soc. Sports Nutr..

[4] Pasiakos S.M., Lieberman H.R., McLellan T.M. (2014). Effects of protein supplements on muscle damage, soreness and recovery of muscle function and physical performance: A systematic review. Sports Med..

[5] Nosaka K., Sacco P., Mawatari K. (2006). Effects of Amino Acid Supplementation on Muscle Soreness and Damage. Int. J. Sport Nutr. Exerc. Metab..

[6] Harris R.A., Joshi M., Jeoung N.H. (2004). Mechanisms responsible for regulation of branched-chain amino acid catabolism. Biochem. Biophys. Res. Commun..

[7] Shimomura Y., Murakami T., Nakai N., Nagasaki M., Harris R.A. (2004). Exercise promotes BCAA catabolism: Effects of BCAA supplementation on skeletal muscle during exercise. J. Nutr..

[8] Coombes J.S., McNaughton L.R. (2000). Effects of branched-chain amino acid supplementation on serum creatine kinase and lactate dehydrogenase after prolonged exercise. J. Sports Med. Phys. Fitness.

[9] Ra S.-G., Miyazaki T., Ishikura K., Nagayama H., Komine S., Nakata Y., Maeda S., Matsuzaki Y., Ohmori H. (2013). Combined effect of branched-chain amino acids and taurine supplementation on delayed onset muscle soreness and muscle damage in high-intensity eccentric exercise. J. Int. Soc. Sports Nutr..

[10] Howatson G., Hoad M., Goodall S., Tallent J., Bell P.G., French D.N. (2012). Exercise-induced muscle damage is reduced in resistance-trained males by branched chain amino acids: A randomized, double-blind, placebo controlled study. J. Int. Soc. Sports Nutr..

[11] Sharp C.P., Pearson D.R. (2010). Amino acid supplements and recovery from high-intensity resistance training. J. Strength Cond. Res..

[12] Greer B.K., Woodard J.L., White J.P., Arguello E.M., Haymes E.M. (2007). Branched-chain amino acid supplementation and indicators of muscle damage after endurance exercise. Int. J. Sport Nutr. Exerc. Metab..

[13] Blomstrand E. (2006). A role for branched-chain amino acids in reducing central fatigue. J. Nutr..

[14] Newsholme E.A., Blomstrand E. (2006). Branched-chain amino acids and central fatigue. J. Nutr..

[15] Jackman S.R., Witard O.C., Jeukendrup A.E., Tipton K.D. (2010). Branched-chain amino acid ingestion can ameliorate soreness from eccentric exercise. Med. Sci. Sports Exerc..

[16] Fouré A., Nosaka K., Gastaldi M., Mattei J.-P., Boudinet H., Guye M., Vilmen C., Le Fur Y., Bendahan D., Gondin J. (2016). Effects of branched-chain amino acids supplementation on both plasma amino acids concentration and muscle energetics changes resulting from muscle damage: A randomized placebo controlled trial. Clin. Nutr..

[17] Areces F., Salinero J.J., Abian-Vicen J., González-Millán C., Gallo-Salazar C., Ruiz-Vicente D., Lara B., Del Coso J. (2014). A 7-day oral supplementation with branched-chain amino acids was ineffective to prevent muscle damage during a marathon. Amino Acids.

[18] Shimomura Y., Murakami T., Nagasaki M., Honda T., Goto H., Kotake K., Kurokawa T., Nonami T. (2004). Regulation of branched-chain amino acid metabolism and pharmacological effects of branched-chain amino acids. Hepatol. Res..

[19] Kreider R.B., Miriel V., Bertun E. (1993). Amino acid supplementation and exercise performance. Analysis of the proposed ergogenic value. Sports Med..

[20] Williams M.H. (1999). Facts and fallacies of purported ergogenic amino acid supplements. Clics Sports Medice.

[21] Jager R., Kerksick C.M., Campbell B.I., Cribb P.J., Wells S.D., Skwiat T.M., Purpura M., Ziegenfuss T.N., Ferrando A.A., Arent S.M. (2017). International society of sports nutrition position stand: Protein and exercise. J. Int. Soc. Sports Nutr..

[22] Morton R.W., Murphy K.T., McKellar S.R., Schoenfeld B.J., Henselmans M., Helms E., Aragon A.A., Devries M.C., Banfield L., Krieger J.W. (2018). A systematic review, meta-analysis and meta-regression of the effect of protein supplementation on resistance training-induced gains in muscle mass and strength in healthy adults. Br. J. Sports Med..

[23] Shimomura Y., Harris R.A. (2006). Metabolism and physiological function of branched-chain amino acids: Discussion of session 1. J. Nutr..

[24] Shimomura Y., Yamamoto Y., Bajotto G., Sato J., Murakami T., Shimomura N., Kobayashi H., Mawatari K. (2006). Nutraceutical effects of branched-chain amino acids on skeletal muscle. J. Nutr..

[25] Greenhaff P.L., Casey A., Short A.H., Harris R., Soderlund K., Hultman E. (1993). Influence of oral creatine supplementation of muscle torque during repeated bouts of maximal voluntary exercise in man. Clin. Sci..

[26] Jackson A.S., Pollock M.L. (1978). Generalized equations for predicting body density of men. Br. J. Nutr..

[27] Siri W.E. (1993). Body composition from fluid spaces and density: Analysis of methods. 1961. Nutrition.

[28] Kraemer W.J., Fry A.C. (1995). ACSM’s Guidelines for Exercise Testing and Prescription.

[29] Thompson B.J., Ryan E.D., Sobolewski E.J., Conchola E.C., Cramer J.T. (2013). Age related differences in maximal and rapid torque characteristics of the leg extensors and flexors in young, middle-aged and old men. Exp. Gerontol..

[30] Hasson C.J., Dugan E.L., Doyle T.L.A., Humphries B., Newton R.U. (2004). Neuromechanical strategies employed to increase jump height during the initiation of the squat jump. J. Electromyogr. Kinesiol..

[31] Dugan E.L., Doyle T.L., Humphries B., Hasson C.J., Newton R.U. (2004). Determining the optimal load for jump squats: A review of methods and calculations. J. Strength Cond. Res..

[32] Baird M.F., Graham S.M., Baker J.S., Bickerstaff G.F. (2012). Creatine-kinase- and exercise-related muscle damage implications for muscle performance and recovery. J. Nutr. Metab..

[33] Nikolaidis M.G., Jamurtas A.Z., Paschalis V., Fatouros I.G., Koutedakis Y., Kouretas D. (2008). The effect of muscle-damaging exercise on blood and skeletal muscle oxidative stress: Magnitude and time-course considerations. Sports Med..

[34] Hyldahl R.D., Hubal M.J. (2013). Lengthening our perspective: Morphological, cellular and molecular responses to eccentric exercise. Muscle Nerve.

[35] Atherton P.J., Kumar V., Selby A.L., Rankin D., Hildebrandt W., Phillips B.E., Williams J.P., Hiscock N., Smith K. (2016). Enriching a protein drink with leucine augments muscle protein synthesis after resistance exercise in young and older men. Clin. Nutr..

[36] Reidy P.T., Rasmussen B.B. (2016). Role of ingested amino acids and protein in the promotion of resistance exercise-induced muscle protein anabolism. J. Nutr..

[37] Morton R.W., McGlory C., Phillips S.M. (2015). Nutritional interventions to augment resistance training-induced skeletal muscle hypertrophy. Front Physiol..

[38] Kirby T.J., Triplett N.T., Haines T.L., Skinner J.W., Fairbrother K.R., McBride J.M. (2012). Effect of leucine supplementation on indices of muscle damage following drop jumps and resistance exercise. Amino Acids.

[39] Shimomura Y., Inaguma A., Watanabe S., Yamamoto Y., Muramatsu Y., Bajotto G., Sato J., Shimomura N., Kobayashi H., Mawatari K. (2010). Branched-chain amino acid supplementation before squat exercise and delayed-onset muscle soreness. Int. J. Sport Nutr. Exerc. Metab..

[40] Ohtani M., Sugita M., Maruyama K. (2006). Amino acid mixture improves training efficiency in athletes. J. Nutr..

[41] Margaritelis N.V., Theodorou A.A., Baltzopoulos V., Maganaris C.N., Paschalis V., Kyparos A., Nikolaidis M.G. (2015). Muscle damage and inflammation after eccentric exercise: Can the repeated bout effect be removed?. Physiol. Rep..

[42] Kamandulis S., Skurvydas A., Brazaitis M., Škikas L., Duchateau J. (2009). The repeated bout effect of eccentric exercise is not associated with changes in voluntary activation. Eur. J. Appl. Physiol..

[43] Meldrum D., Cahalane E., Keogan F., Hardiman O. (2003). Maximum voluntary isometric contraction: Investigation of reliability and learning effect. Amyotroph. Lateral. Scler. Other Motor Neuron Disord..

[44] Warren G.L., Lowe D.A., Armstrong R.B. (1999). Measurement tools used in the study of eccentric contraction-induced injury. Sports Med..

[45] Cheung K., Hume P., Maxwell L. (2003). Delayed onset muscle soreness: Treatment strategies and performance factors. Sports Med..

[46] Lewis P.B., Ruby D., Bush-Joseph C.A. (2012). Muscle soreness and delayed-onset muscle soreness. Clin. Sports Med..

[47] Deyhle M.R., Gier A.M., Evans K.C., Eggett D.L., Nelson W.B., Parcell A.C., Hyldahl R.D. (2016). Skeletal muscle inflammation following repeated bouts of lengthening contractions in humans. Front. Physiol..

[48] Nicastro H., da Luz C.R., Chaves D.F.S., Bechara L.R.G., Voltarelli V.A., Rogero M.M., Lancha A.H. (2012). Does branched-chain amino acids supplementation modulate skeletal muscle remodeling through inflammation modulation? Possible mechanisms of action. J. Nutr. Metab..

[49] Greer B.K., White J.P., Arguello E.M., Haymes E.M. (2011). Branched-chain amino acid supplementation lowers perceived exertion but does not affect performance in untrained males. J. Strength Cond. Res..

[50] Howatson G., van Someren K.A. (2007). Evidence of a contralateral repeated bout effect after maximal eccentric contractions. Eur. J. Appl. Physiol..

[51] Ratamess N.A., Kraemer W.J., Volek J.S., Rubin M.R., Gómez A.L., French D.N., Sharman M.J., McGuigan M.M., Scheett T., Häkkinen K. (2003). The effects of amino acid supplementation on muscular performance during resistance training overreaching. J. Strength Cond. Res..

[52] Ohtani M., Kawada S., Seki T., Okamoto Y. (2012). Amino acid and vitamin supplementation improved health conditions in elderly participants. J. Clin. Biochem. Nutr..

[53] Marg A., Schoewel V., Timmel T., Schulze A., Shah C., Daumke O., Spuler S. (2012). Sarcolemmal repair is a slow process and includes ehd2. Traffic.

[54] Hyldahl R.D., Chen T.C., Nosaka K. (2017). Mechanisms and Mediators of the Skeletal Muscle Repeated Bout Effect. Exerc. Sport Sci. Rev..

